# ORF3a mutation associated with higher mortality rate in SARS-CoV-2 infection

**DOI:** 10.1017/S0950268820002599

**Published:** 2020-10-26

**Authors:** Parinita Majumdar, Sougata Niyogi

**Affiliations:** Independent Researcher

**Keywords:** COVID-19, ORF3a, mortality, mutation, SARS-CoV-2

## Abstract

Severe acute respiratory syndrome coronavirus 2 (SARS-CoV-2) has recently caused acute respiratory distress syndrome affecting more than 200 countries with varied mortality rate. Successive genetic variants of SARS-CoV-2 become evident across the globe immediately after its complete genome sequencing. Here, we found a decent association of SARS-CoV-2 ORF3a mutation with higher mortality rate. Extensive *in silico* studies revealed several amino acid changes in ORF3a protein which ultimately leads to diverse structural modifications like B cell epitope loss, gain/loss of phosphorylation site and loss of leucine zipper motif. We could further relate these changes to the enhanced antigenic diversity of SARS-CoV-2. Through protein−protein network analysis and functional annotation studies, we obtained a close federation of ORF3a protein with host immune response *via* divergent signal transduction pathways including JAK-STAT, chemokine and cytokine-related pathways. Our data not only unveil the fairly appreciable association of ORF3a mutation with higher mortality rate, but also suggest a potential mechanistic insight towards the immunopathogenic manifestation of SARS-CoV-2 infection.

## Graphical abstract

**Figure:**
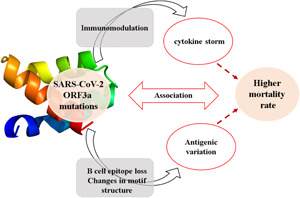

## Introduction

The betacoronaviruses of *Coronaviridae* family are potent human pathogens of zoonotic origin [1, 2]. Till now, human coronaviruses (CoVs) have been associated with three major outbreaks of acute respiratory disorders namely severe acute respiratory syndrome (SARS) in 2002, middle-east respiratory syndrome (MERS) in 2012 and COVID-19 in 2019 (Coronavirus disease 2019) with an overall mortality rate of 9.6%, 40% and 6.9%, respectively [1, 3-5, https://www.worldometers.info/coronavirus/]. COVID-19 originated at Wuhan city of Central China in mid-November 2019, gradually spread to more than 200 countries with a death toll of 2,44,664 as on 2 May 2020 [5, https://www.worldometers.info/coronavirus/]. The complete genome sequence of the causative organism was first reported from China and found to be severe acute respiratory syndrome coronavirus 2 (SARS-CoV-2) which bears 79% and 50% genetic similarities with SARS-CoV and MERS-CoV [2, 5]. The unusually larger, ~30 kb genome of SARS-CoV-2 has similar genomic organisation with SARS-CoV and encodes 16 non-structural proteins (NSP1−NSP16), 4 structural proteins including Spike (S), Membrane (M), Envelope (E) and Nucleocapsid (N) proteins and 6 accessory proteins (ORF3a, ORF6, ORF7a, ORF7b, ORF8 and ORF10) [2, 4-6]. The non-structural proteins of CoVs are indispensable for viral replication and transcription whereas the structural proteins constitute the mature virion [2, 5]. The role of accessory proteins has just begun to be evident in viral pathogenesis [7-9]. Among the accessory proteins, ORF3a is the largest one containing 274 amino acids in SARS-CoV [9]. Cell surface localisation of ORF3a in SARS-CoV potentiates viral entry within the host and has immunogenic properties [3, 9]. Moreover, it is involved in pro-inflammatory cytokine and chemokine production by activating various signal transduction pathways including C-Jun N-terminal kinase (JNK) and nuclear factor kappa-light-chain-enhancer of activated B cells (NF-*κ*B) [10]. An uncontrolled release of pro-inflammatory cytokines and chemokines results in an exacerbated host immune response in SARS-CoV-2 infected patients leading to cytokine storm [11]. ORF3a is also implicated in ion channel formation and modulates release of virus from the host cell [9].

Soon after the isolation of Wuhan strain (EPI_ISL_402123), an increasing number of SARS-CoV-2 variants with genetic diversity has started to emerge across the globe [4-6, 12]. The large genome size of SARS-CoV-2 and low fidelity RNA-dependent RNA polymerase (RdRp) are the contributing factors for considerable higher rate of adaptive mutations leading to the evolution and coexistence of pathogenic viral strains worldwide [4, 6, 13]. Several independent recurrent mutations in NSP6, NSP7, NSP12, NSP13 encoded by ORF1ab and spike protein resulting in non-synonymous amino acid mutations are identified as mutational hotspot which are closely associated with inter-species transmission and virulence [4, 6, 14-15]. Thus it is intriguing to understand the functional implications of different mutations in the diverse infection and mortality rate of SARS-CoV-2.

## Methods

### Study design and data collection

**Fig. 1.**
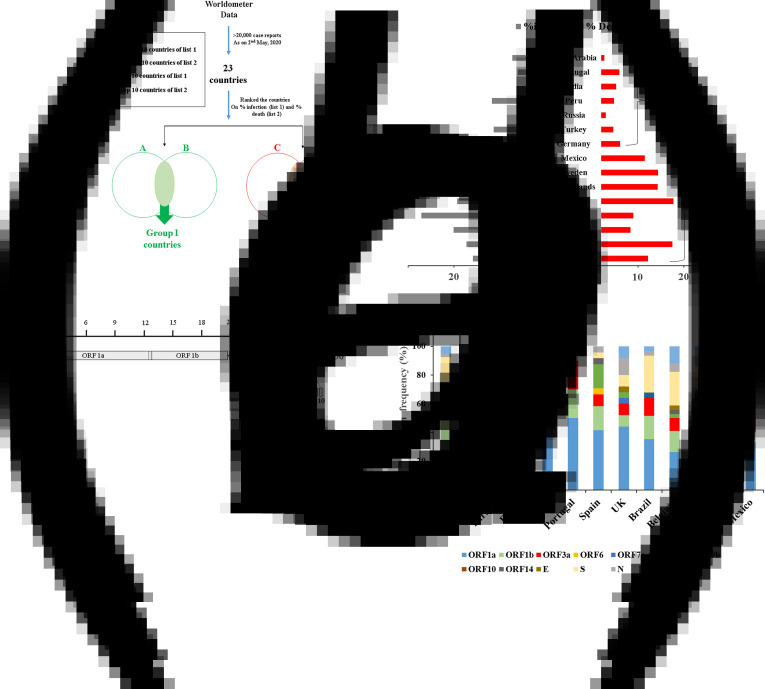
SARS-CoV-2 ORF3a mutations is associated with higher infection and mortality rate: (a) Overall screening scheme of the study. (b) Tornedo plot showing differential infection and death rate (%) of COVID-19 for indicated countries. (c) Genomic organisation of SARS-CoV-2 depicting protein coding ORFs. (d) Stacked bar chart showing mutation frequency at indicated ORFs for respective countries.

All data were collected from coronavirus worldometer section (https://www.worldometers.info/coronavirus/) as on 2 May 2020. Initially, 23 countries were selected with a minimum positive 20,000 case reports. Percent infection was defined as (total cases/total tests) × 100 and % death was calculated as (total deaths/total cases) × 100 (online Supplementary table 1). Fifteen countries with either lower (group I) or higher (group II) values (both % infection and % death) were shortlisted for further analysis (Fig. 1a and b). Wuhan strain from China (EPI_ISL_402123) was included as a reference for most of the analysis.

### Mutation frequency calculation

Amino acid changes in SARS-CoV-2 strains for each country were extracted from NextStrain open source project (https://nextstrain.org/ncov) [12]. Mutation frequency was calculated by dividing the number of mutations for a particular protein with total number of mutations corresponding to all the proteins for a given country, multiplied by 100. The mutation frequency was represented by stacked bar chart which was created using Microsoft Excel.

### Sequence alignment

Amino acid sequences of ORF3a protein (NP_828852.2 and YP_009724391.1 for SARS-CoV and SARS-CoV-2, respectively) in FASTA format were downloaded from NCBI protein database and pair-wise sequence alignment was performed in EMBOSS Needle web server with default parameters [16].

### Epitope and biological function prediction

Amino acid sequences of concerned proteins were given to IEDB-AR (Immune Epitope Database Analysis Resource) for B cell epitope prediction using default parameters and 0.55 threshold value [17]. Biological significance of any mutation was determined using PROVEAN web tool (v1.1.3). FASTA sequence of a wild-type query protein and mutant variants were uploaded. Functional relevance was predicted with default settings [18].

### Protein structure prediction

Amino acid sequences of ORF3a protein (NP_828852.2 and YP_009724391.1 for SARS-CoV and SARS-CoV-2, respectively) in FASTA format were further scanned in ExPASy**-**prosite (prosite.expasy.org) portal for motif analysis. Tertiary structures for ORF3a protein were obtained from Phyre2 web portal upon amino acid sequence submission (FASTA).

### Viral protein interactome study

Human interacting partners of SARS-CoV ORF3a protein were charted using STRING Viruses database (10.5). Not more than 20 1^st^ shell and <10 2^nd^ shell interactions were taken with a threshold confidence score of 0.7 [19].

### Functional annotation and pathway prediction

Twelve different human interacting partners of ORF3a protein were taken for functional annotation in DAVID (Database for Annotation, Visualization and Integrated Discovery, v6.8) [20]. After gene-enrichment analysis, functional annotation clustering and KEGG (Kyoto encyclopedia for gene and genomes) pathway mapping, a cluster with highest enrichment score (4.11) was obtained.

## Results

### Mutation in ORF3a protein of SARS-CoV-2 is associated with higher infection and mortality rate

Initially 23 countries with more than 20,000 COVID-19 positive case reports were selected for analysis. These countries were ranked based on percent infection (list1) and percent death (list 2) (Fig. 1a). A and B represent bottom ten countries from list 1 and 2, respectively. While C and D correspond to top ten countries from those two lists. Now these countries were categorised into two groups; low infection as well as mortality rate (group I) and high infection and mortality rate (group II) (Fig. 1a). Based on these parameters, 15 countries were further short-listed where Saudi Arabia, Portugal, India, Peru, Russia, Turkey and Germany fell into group I while Mexico, Sweden, Netherlands, Belgium, Brazil, Iran, UK and Spain belonged to group II (Fig. 1b, online Supplementary table 1). A total of 218 viral strains from 15 countries (including China as reference) were further analysed for amino acid mutations from NextStrain database (online Supplementary table 2). Saudi Arabia, Peru and Iran were excluded from the analysis due to inadequate data. Mutations were found in almost all the ORFs including ORF1a, 1b, 3a, 6, 7a, 8, 10, E, S, N and M with varying frequency (Fig. 1c and d). Mutations with considerably higher frequency was observed in ORF1a and ORF1b which comprise two-third of the viral genome and was prevalent in all group I and group II countries (Fig. 1d). Similarly, mutation in S protein was common in all the countries tested except Portugal. Some of the group II countries including UK, Brazil, Belgium and Netherlands exhibited mutations in M protein which was completely absent from group I countries (Fig. 1d). Further, viral strains from all the countries except Germany accumulated mutations in N protein indicating a probable mutational hotspot. Interestingly, ORF3a mutation was present in all group II countries which were not found in group I (except Portugal). Based on the rapid emergence of multiple genetic variants, we speculate a potential involvement of SARS-CoV-2 genetic diversity in varied clinical outcomes of its infection. Thus along with diverse regulating factors such as age, sex, co-morbidity complications like diabetes, cardiovascular diseases and chronic lung diseases, ORF3a mutations are linked with differential infection and mortality rate of SARS-CoV-2 infection (Fig. 1b).

### Biological significance of ORF3a mutations in SARS-CoV-2

**Fig. 2.**
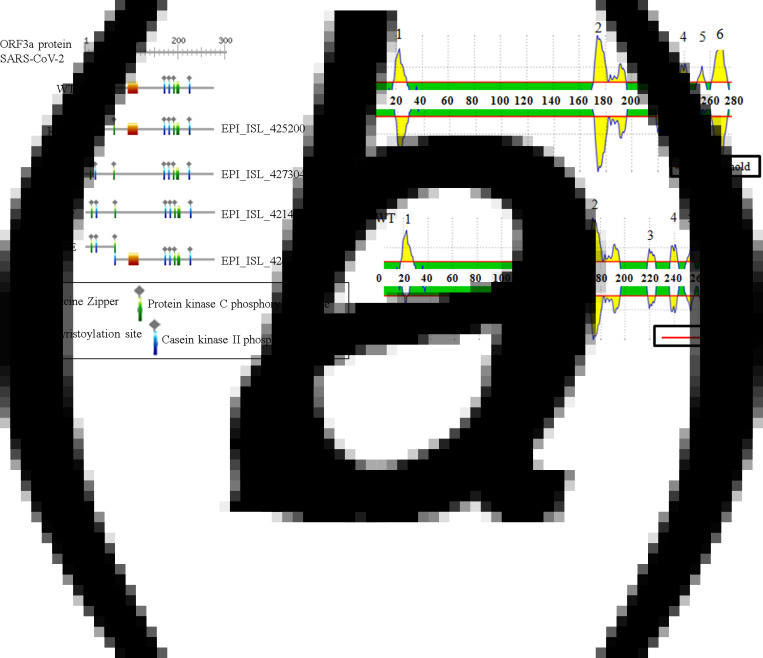
Biological relevance of ORF3a mutations in SARS-CoV-2: (a) ExPASy-Prosite motif prediction data of ORF3a proteins for wild-type and indicated mutant variants of SARS-CoV-2. (b) B cell epitope prediction data for wild-type and mentioned mutant variant of SARS-CoV-2. Arrow indicates epitope loss.

The association between ORF3a mutations and countries with higher infection as well as mortality rate instigated us to elucidate the nature of amino acid mutations in each of the viral isolates. Altogether, 18 different amino acid changes were observed of which 5 mutations (T175I, L94F, K16N, L94I and A72 T) were neutral and remaining were deleterious (Q57H, G251 V, P25L, W149L, R126 T, T176I, T217I, D142N, V90F, Y109C, D155Y, Y156N and K67E) (online Supplementary Table 3). Some of these mutations resulted in the loss of predicted motifs and B cell epitope as found in wild-type (WT) ORF3a protein (Fig. 2). The WT ORF3a protein from SARS-CoV-2 was predicted to have N-myristoylation site, leucine-zipper and phosphorylation sites for protein kinase C as well as casein kinase II, (Fig. 2a). K16N (EPI_ISL_425200) and K67E (EPI_ISL_424731) mutations resulted in the loss and gain of phosphorylation sites in ORF3a variants from Spain and Mexico, respectively (Fig. 2a). L94I (EPI_ISL_427304) and L94F (EPI_ISL_421460) mutations led to the loss of leucine-zipper from Brazil and Portugal isolates. WT ORF3a protein is also predicted to have six B cell epitopes with varying intensities spanning from 19 to 28 (1), 172−196 (2), 219−225 (3), 237−243 (4), 251−256 (5) and 261−273 (6) amino acids (Fig. 2b, upper panel). Interestingly, G251 V (EPI_ISL_428674) mutation resulted in the loss of B cell epitope corresponding to 251−256 amino acid residues. P25L mutation accounted for the drop in epitope intensity encompassing 19−28 position. However, there was no change in the epitope number for this variant (Fig. 2b, lower panel). Thus changes in amino acid residues are likely to interfere with the biological role of WT SARS-CoV-2 ORF3a protein and needs to be explored in context of host−pathogen interaction.

### ORF3a protein of SARS-CoV-2 structurally resembles ORF3a of SARS-CoV

**Fig. 3.**
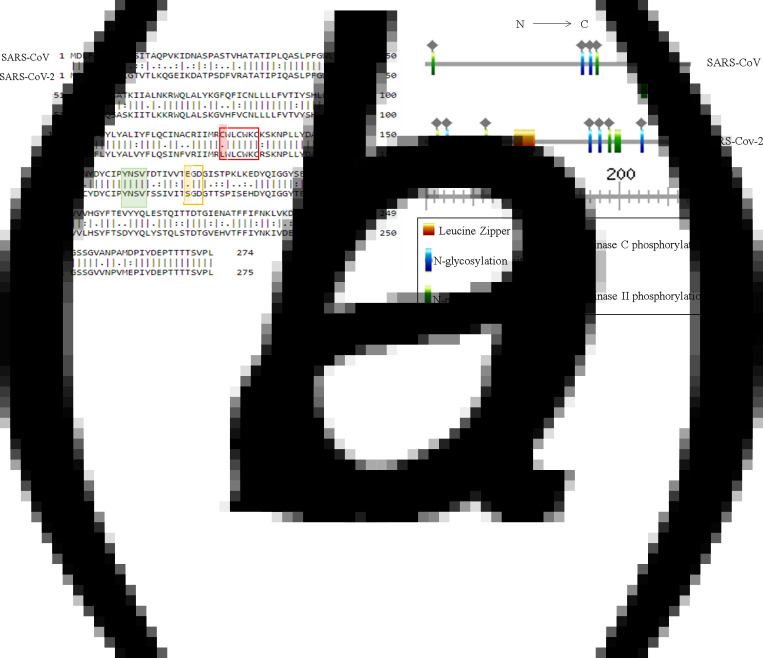
Structural comparison between ORF3a proteins of SARS-CoV and SARS-CoV-2: (a) Pair-wise sequence alignment of SARS-CoV and SARS-CoV-2 ORF3a proteins. Red, green and yellow boxes denote cysteine, YxxΦ and di-acidic motifs, respectively. Change in amino acid residues are highlighted in red and yellow. **(b)** ExPASy**-**Prosite motif prediction data of SARS-CoV and SARS-CoV-2 ORF3a proteins.

Putative ORF3a of SARS-CoV-2 is predicted to encode 275 amino acids containing protein of 31 kDa molecular weight [8]. SARS-CoV-2 ORF3a bears 72.4% sequence identity and 85.1% sequence similarity with that of SARS-CoV (Fig. 3a). Currently, crystal structures of SARS-CoV and SARS-CoV-2 ORF3a are not yet resolved. However, the predicted tertiary structures of ORF3a protein from SARS-CoV exhibited 3 alpha helices which were also observed in SARS-CoV suggesting functional conservation (Supplementary Fig. 1a and b). ORF3a protein of SARS-CoV has three N terminal transmembrane domains and exhibits both plasma membrane and intracellular localisations [7, 9]. SARS-CoV-2 ORF3a is also predicted to have N terminal transmembrane domains similar to SARS-CoV ORF3a (Supplementary Fig. 1c and d). *In silico* motif scan showed the presence of conserved N-myristoylation site and several phosphorylation sites for protein kinase C and casein kinase II in SARS-CoV and SARS-CoV-2 ORF3a (Fig. 3b). However, a leucine zipper was predicted only in SARS-CoV-2 while N-glycosylation site was absent which was predicted in SARS-CoV ORF3a (Fig. 3b). SARS-CoV ORF3a protein has been shown to have cysteine motif CWLCWKC (127−133 amino acid), di-acidic motif ExD (171−173 amino acid) and C terminal YxxΦ domain (160−163 amino acid), which are implicated in mediating protein−protein interactions with other SARS-CoV proteins, export from endoplasmic reticulum and host immune evasion [9]. Sequence alignment of SARS-CoV and SARS-CoV-2 ORF3a proteins revealed C127L mutation in cysteine motif of SARS-CoV-2 (Fig. 3a) and E171S mutation in di-acidic motif (Fig. 3a). However, YxxΦ domain was conserved between the two proteins (Fig. 3a marked in green).

### ORF3a protein of SARS-CoV-2 can potentiate viral immune-pathogenicity

**Fig. 4.**
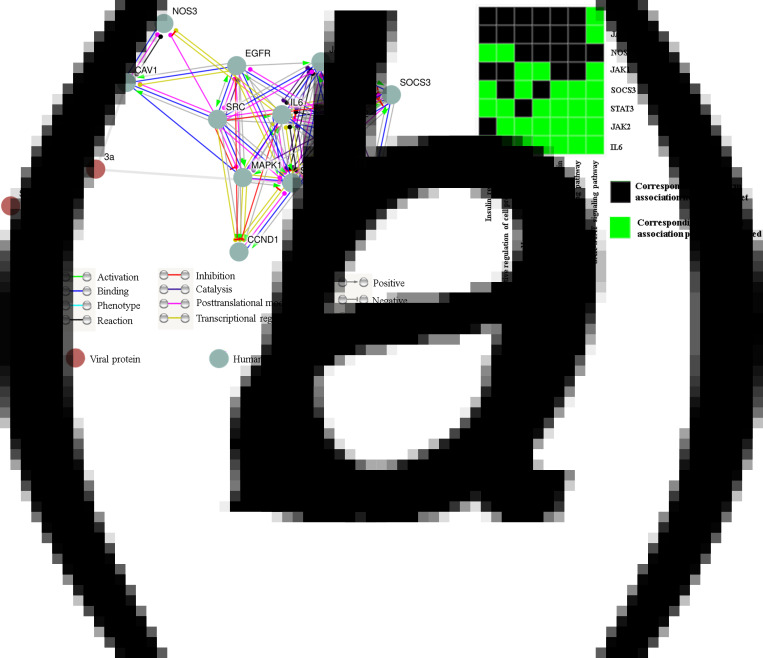
ORF3a protein can influence host immune response: (a) STRING viruses derived ORF3a-host−protein interactions. (b) Functional cluster enrichment analysis of ORF3a interacting partners.

Since none of the accessible SARS-CoV-2 genomes are streamlined in protein databases like STRING we could not directly get ORF3a (SARS-CoV-2) − human protein interactome. Our data have significantly established the structural resemblance of ORF3a protein between SARS-CoV and SARS-CoV-2 thus conceding further functional prediction. So we deduce the functional pertinence of SARS-CoV-2 putative ORF3a protein from interactome and pathway enrichment analysis of SARS-CoV. First, we sought to perform a viral and host protein−protein network analysis using STRING viruses database and found 12 human (JAK1/2/3, STAT3, SOCS3, NOS3, IL-6, MAPK1, CCND1, CAV1, SRC and EGFR) and one viral (S glycoprotein) interacting partners (direct or indirect) for ORF3a protein of SARS-CoV. Only two host proteins (STAT3 and CAV1) were acquired as direct interacting partners (Fig. 4a). Next, we used this list of interacting protein encoding genes in DAVID for functional annotation and pathway prediction. We got disparate pathways like TLR signalling pathway, NOD like receptor pathway, TNF signalling pathway and T cell receptor pathway to be associated with this ORF3a interacting nexus (online Supplementary Table 4). These pathways were mostly connected to immune response and signal transduction suggesting potential involvement of SARS-CoV-2 ORF3a protein in host immune modulation. Surprisingly, insulin resistance was found to be linked with this ORF3a interactome. PI3 K-Akt signalling pathway, chemokine pathway and JAK-STAT pathway were present for almost all the interacting partners (online Supplementary Table 4). A direct interacting host protein CAV1 was found to be federated with endocytic pathways indicating probable contribution of ORF3a towards viral entry (Fig. 4a, online Supplementary Table 4). Other intracellular signalling pathways such as VEGF, MAPK, AMPK pathways were detected to be linked to this interacting network (online Supplementary Table 4). A cluster of 8 interacting partners (STAT3, JAK1/2/3, NOS3, SOCS3, IL-6, CCND1) were given highest enrichment score of 4.11. In ‘X’ axis of the cluster 7 different associated pathways (JAK-STAT signalling pathway, cytokine-mediated signalling pathway, positive regulation of tyrosine phosphorylation of STAT3 protein, IL6 type cytokine signal transduction, Herpes simplex infection, negative regulation of cell proliferation and insulin resistance) are shown (Fig. 4b). IL-6 was reported to be federated with all the charted pathways whereas JAK3 and CCND1 were found to be linked with only JAK-STAT pathway. While JAK2 and STAT3 were associated with all the noted pathways except insulin resistance and Herpes simplex infection, respectively, only two connections (negative regulation of cell proliferation and insulin resistance) were reported for NOS3 (Fig. 4b). Interestingly, all the proteins were somehow linked to JAK-STAT pathway.

## Discussion

Since its discovery, seven human CoVs have been delineated and the recent one, SARS-CoV-2 engendered a global medical emergency [1-2]. Even after contemplating several climatic, anthropometric and clinical factors [4], we found an unforeseen variation in the mortality rate among different countries. These countries were categorised into two distinct groups depending on their infection and mortality rate (Fig. 1a-b). There are persuasive mathematical models suggesting evolutionary and causal relationships between virulence and mortality rate [21]. Virulence evolution is further affected by intra-host mutation and inter-strain competition-mediated selection pressure of an obligate parasite like virus [22]. Human SARS-CoV has a fairly large and flexible genome thus allowing considerable amount of adaptive mutations further leading to the emergence of novel strains with enhanced pathogenicity [23]. In agreement to this we could also detect various mutations (amino acid changes) in 13 different ORFs of SARS-CoV-2 and interestingly ORF3a mutations were found to be markedly associated with group II countries having higher mortality rate (Fig. 1d). Where the changes in phosphorylation sites in ORF3a of SARS-CoV-2 variants might be responsible for functional involvement in signal transduction pathways, N-myristoylation site remained conserved indicating the intact viral immune-pathogenicity [24]. K67E variant showed gain of casein kinase II phosphorylation site. Computer-aided study revealed N protein of coronavirus as a probable substrate of casein kinase II [25] but no studies are available to suggest any possible aftermath of a casein kinase phosphorylation site gain. Leucine zipper motif loss in murine coronavirus S protein could impart defective cell to cell fusion [26] but potential implication of such mutation in ORF3a protein of human corona virus remained elusive. The N terminal peptide of SARS-CoV-2 ORF3a protein spanning from 15 to 28 amino acids had been shown to induce strong and protective humoral response in the infected patient [27]. We found a drastic drop in B cell epitope intensity of SARS-CoV-2 variants (P25L) which suggests a probable mechanism of immune evasion and its enhanced virulence. However, the significance of the loss of B cell epitope in G251 V variant needs to be addressed. Through sundry *in silico* approaches we could show appreciable structural similarities of SARS-CoV and SARS-CoV-2 ORF3a proteins, suggesting their functional accordance (Fig. 3a-b, online Supplementary Fig. 1a-d). Although these *in silico* predictions warrant further *in vivo* validations. ORF3a protein was extensively implicated in host immune evasion, immunomodulation and virulence of SARS-CoV *via* NF-*κ*B, JNK and cytokine pathways [7]. In addition to virulence and pathogenecity, ORF3a protein of SARS-CoV-2 is predicted to be involved in ion channel formation and viral release, similar to SARS-CoV ORF3a [8]. Our study did not find any mutations in the functional motifs of SARS-CoV-2 ORF3a involved in such functions (Fig. 3a). Thus it is unlikely that these mutations would affect viral life cycle. However, the precise role of ORF3a protein in SARS-CoV-2 life cycle needs further investigation.

Our functional enrichment data also stipulate the reasonable association of ORF3a protein with host immune response through JAK-STAT, chemokine and cytokine-related pathways (Fig. 4b and online Supplementary Table 4). Together we could sensibly propose the potential role of ORF3a mutations in elevated mortality rate for SARS-CoV-2 infection through host immune evasion and immoderate cytokine storm.

## Data Availability

The data integral to this finding would be available from the corresponding author upon request. The mutation data on various SARS-CoV-2 proteins are freely accessible from NextStrain open source project (https://nextstrain.org/ncov).

## Supplementary material

For supplementary material accompanying this paper visit http://dx.doi.org/10.1017/S0950268820002599. click here to view supplementary material
