# Supplementary material for: ORF3a mutation associated with higher mortality rate in SARS-CoV-2 infection
Source: Epidemiol Infect. 2020 Oct 26;148:e262. doi: 10.1017/S0950268820002599 (PMC7653495; doi:10.1017/S0950268820002599)
Supplement: Supplementary file 1 [file S0950268820002599sup001.docx]

**Supplementary Material**

**ORF3a mutation associated higher mortality rate in SARS-CoV-2 infection**

Parinita Majumdar and Sougata Niyogi*

*Correspondence

Dr. Sougata Niyogi

E mail: [sniyogi10@gmail.com](mailto:sniyogi10@gmail.com)

Phone: +91 8240983494

**Supplementary Figure Legends**

**Supplementary Figure 1: Tertiary structure of ORF3a protein from SARS-CoV and SARS-CoV-2:** Predicted tertiary structures of ORF3a protein for SARS-CoV **(a)** and SARS-CoV-2 **(b)**. Transmembrane organizations of ORF3a protein for SARS-CoV **(c)** and SARS-CoV-2 **(d)**.

**Supplementary** **Table Legends**

**Supplementary Table 1:** The percent infection and death of COVID-19 patients from indicated countries.

**Supplementary Table 2:** Country wise list of different amino acid mutations in SARS-CoV-2 proteins.

**Supplementary Table 3:** Divergent amino acid mutations in ORF3a protein of SARS-CoV-2.

**Supplementary Table 4:** List of different pathways related to ORF3a protein interactome.

**Supplementary Figure 1**


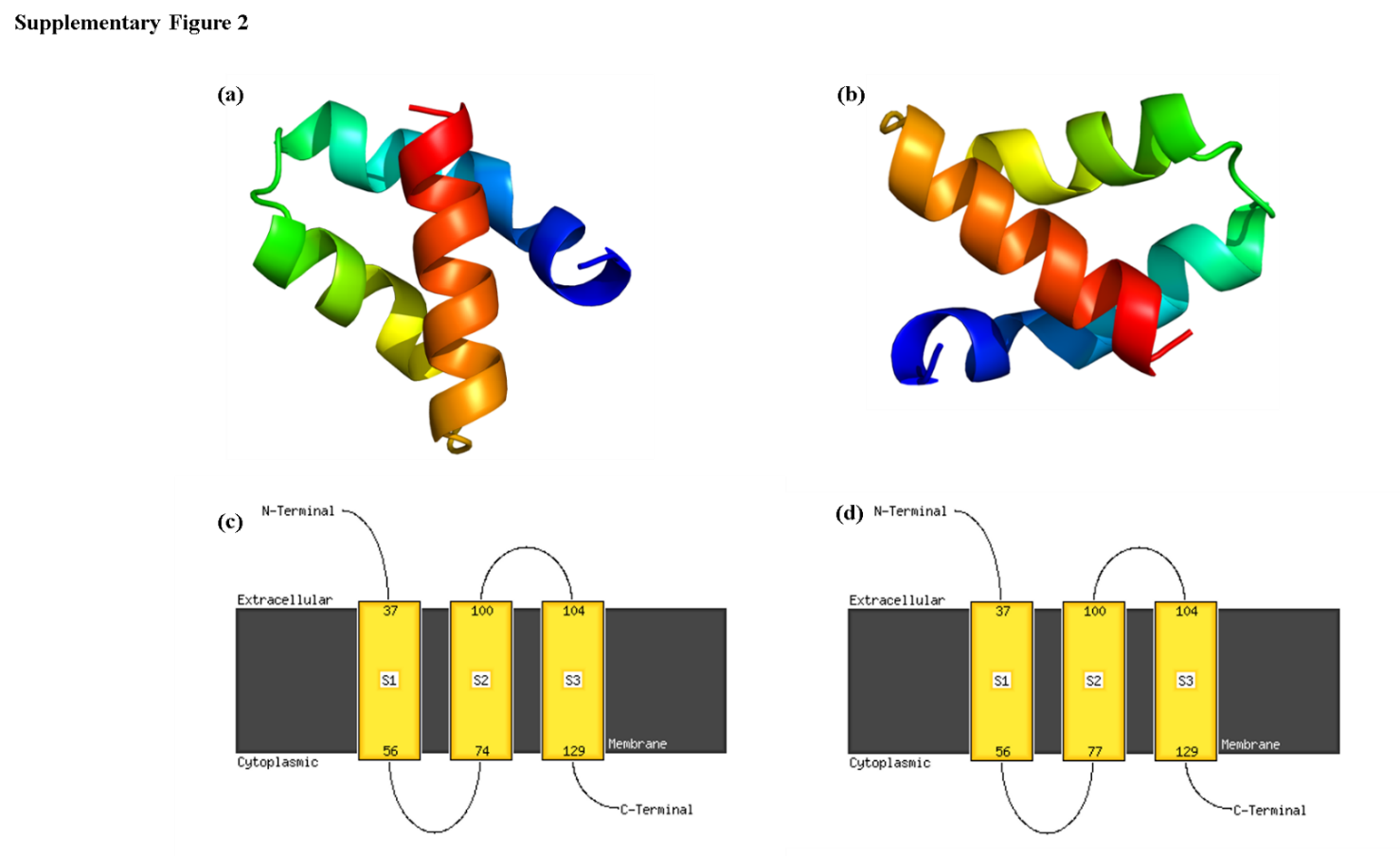


**Supplemental Table 1**

| Country | %Infection | % Death |
| --- | --- | --- |
|  |  |  |
| Spain | 15.89369146 | 10.21614236 |
| UK | 17.33247121 | 15.50260913 |
| Iran | 20.13502504 | 6.36827468 |
| Brazil | 27.1540147 | 6.954296002 |
| Belgium | 19.36508187 | 15.71014847 |
| Netherlands | 17.61450914 | 12.29675052 |
| Sweden | 18.0083682 | 12.32806691 |
| Mexico | 22.7431241 | 9.508655191 |
| Germany | 6.441839428 | 4.105389543 |
| Turkey | 11.38479398 | 2.661938689 |
| Russia | 3.09272973 | 1.021576321 |
| Peru | 11.8129157 | 2.778121061 |
| India | 3.823987595 | 3.275658882 |
| Portugal | 6.183758943 | 3.972229892 |
| Saudi Arabia | 7.370127388 | 0.701332116 |

| China  **Supplemental Table 2** |  |  |  |  |  |
| --- | --- | --- | --- | --- | --- |
| Proteins | **Amino acid mutation** |  |  |  |  |
| ORF1a | V1994D, G1307S, T3058S, I2244T, D1127V, T2791A, A2001T, N2708S, F2908I |  |  |  |  |
| ORF1b | D2020N, L314P, V658I, A1219S, N30Y, P1001S, V2060A, P1161L, E913G,  L1504I, D1130G, D815Y | |  |  |  |
| ORF3a | Q57H |  |  |  |  |
| ORF7a | Q62*, E92D, *122G, M1R, E121* |  |  |  |  |
| ORF8 | Q91K |  |  |  |  |
| ORF14 | V40F |  |  |  |  |
| S | G614D, S254F, E1258D, R682Q, H49Y, S967F, P970S, K558N |  |  |  |  |
| N | S193I |  |  |  |  |
| M | C64Y, S4F |  |  |  |  |
| Germany |  |  |  |  |  |
| ORF1a | E2993D, T4164I, N3609Y, T2872M, S212L |  |  |  |  |
| ORF1b | A1326S, A1291S, P2321L |  |  |  |  |
| ORF7a | Q62* |  |  |  |  |
| ORF10 | D31Y |  |  |  |  |
| S | E780Q |  |  |  |  |
| Turkey |  |  |  |  |  |
| ORF1a | T951I, A1420V, T4159I, L4182F, G227S, S391F, S911F, L3606F |  |  |  |  |
| ORF1b | G219C, G2662V |  |  |  |  |
| ORF8 | Q72H |  |  |  |  |
| ORF14 | V49I |  |  |  |  |
| S | V772I, T1238I |  |  |  |  |
| N | S202N |  |  |  |  |
| Russia |  |  |  |  |  |
| ORF1a | S674N, T1881I, N1995D, P892S, S3885F, T4129I, T4364I, K2511N, A2994V,  N1576T, G4244R | |  |  |  |
| ORF1b | F1955C, M2269I, P1095S, L1701F, H286Y, V1793I |  |  |  |  |
| ORF6 | P57L |  |  |  |  |
| ORF7a | L5F, A8S |  |  |  |  |
| ORF8 | R101L, G66S |  |  |  |  |
| S | P384L, G261V, D138H |  |  |  |  |
| N | N140K, A397V, A152S, A381V |  |  |  |  |
| Peru |  |  |  |  |  |
| ORF1a | N2894D |  |  |  |  |
| India |  |  |  |  |  |
| ORF1a | K2016T, E381D, E1293D, P1472L, A3615V, I476V, G2035E, V2586G,  V2586G, R3069T, I671T, P2144S, G2118C, D4165E, L2781P,Y4379*,  C4381W, L446I, T592I, V2586G, S1189T | | | | |
| ORF1b | S1089A, A2132T, D2090Y, T1137I, M1397V, A397V, V2475G, A1643V,  P1427L, V871I | |  |  |  |
| ORF6 | E55* |  |  |  |  |
| ORF8 | F120L, I121L |  |  |  |  |
| ORF14 | Q41* |  |  |  |  |
| S | A930V, S943T, R408I, C1250F, T723I, C432*, I434K, T1077S |  |  |  |  |
| N | S194L |  |  |  |  |
| Portugal |  |  |  |  |  |
| ORF1a | R24C, G1261D, I3944M, K1689N, L3800F |  |  |  |  |
| ORF1b | T2592I, P1936S |  |  |  |  |
| ORF3a | T175I, L94F |  |  |  |  |
| N | A217V |  |  |  |  |
| Spain |  |  |  |  |  |
| ORF1a | V424I, L1270F, T3461I, E539D, T2648I, S911F, V3660A, E1047D, L3606F, A1397V |  |  |  |  |
| ORF1b | S2599F, S1273L, T1162I, S358P |  |  |  |  |
| ORF3a | G251V, K16N |  |  |  |  |
| ORF6 | T21I |  |  |  |  |
| ORF8 | P30S, R52T, E106Q, A65V |  |  |  |  |
| ORF14 | Q20R |  |  |  |  |
| S | Q954K |  |  |  |  |
| N | T4171 |  |  |  |  |
| UK |  |  |  |  |  |
| ORF1a | T1000A, T2906A, A3995V, Y3502C, I476V, H83Y, L204F, N3537D, T1093I,  A3755T, P271S | |  |  |  |
| ORF1b | S2363F, A440V |  |  |  |  |
| ORF3a | P25L, W149L |  |  |  |  |
| ORF7a | Y20N |  |  |  |  |
| ORF8 | I9T |  |  |  |  |
| E | L73F |  |  |  |  |
| S | H655Y, M1229I |  |  |  |  |
| N | H300Y, P151L, A156S |  |  |  |  |
| M | R158C, I52T |  |  |  |  |
| Iran |  |  |  |  |  |
| ORF1b | T16371 |  |  |  |  |
| Brazil |  |  |  |  |  |
| ORF1a | T708I, L1599F, F2387L, V1798E, I3108T, G3334S, V378I, Y2384*, L3930F,  T1246I, S3570R | |  |  |  |
| ORF1b | T808R, S2430G, S1661I, A1643G, L627I |  |  |  |  |
| ORF3a | R126T, T176I, L94I, A72T |  |  |  |  |
| ORF9b | P3L |  |  |  |  |
| S | F43L, C1236S, E1207K, S46L, K776T, L938F, A684V, M731I |  |  |  |  |
| N | I292T |  |  |  |  |
| M | I76F |  |  |  |  |
| Belgium |  |  |  |  |  |
| ORF1a | Y4379*, E632G, N1262T, T1454K, L681F, L204F, H3076Y, S3099L |  |  |  |  |
| ORF1b | P909S, A440V, P1879S, P821S, I1250V |  |  |  |  |
| ORF3a | Q57H, T217I, D142N |  |  |  |  |
| ORF8 | P36S |  |  |  |  |
| ORF14 | S18A |  |  |  |  |
| E | T9I |  |  |  |  |
| S | E516Q, K1191N, A771V, K458R, H519P, T941A, L5F, V42L |  |  |  |  |
| N | F171C, D3N |  |  |  |  |
| M | T7I, F193L, T175M, M175T |  |  |  |  |
| Netherlands |  |  |  |  |  |
| ORF1a | T2300I, T4129I, A1997V, V3420I, P3613S, I2538S, L960F, S1468F, G645S,  S2303F, N2187T, L4365F | |  |  |  |
| ORF1b | C1732F, Q866H, F1099L, D870Y, P2116S, I2262V |  |  |  |  |
| ORF3a | Q57H |  |  |  |  |
| ORF9b | R32C |  |  |  |  |
| S | M1237I, T95I, N354K, R765L, I1169T |  |  |  |  |
| N | A35V, K373N, K370N |  |  |  |  |
| M | G78C |  |  |  |  |
| Sweden |  |  |  |  |  |
| ORF1a | V4216F, A3454V, V1211F, I1398V, H3580D, E1210G, S2396I,  A1298V, D1282N |  |  |  |  |
| ORF1b | A1895V, I2465V, M2414I, T17I, A1225S, V248I |  |  |  |  |
| ORF3a | V90F, Y109C, D155Y, Y156N |  |  |  |  |
| ORF7a | R78H, A105S |  |  |  |  |
| ORF8 | A65V |  |  |  |  |
| ORF9b | A29T |  |  |  |  |
| ORF14 | V40F |  |  |  |  |
| S | D936Y, D80Y, V62F, C1243F |  |  |  |  |
| N | R32H, S193I, A280S |  |  |  |  |
| Mexico |  |  |  |  |  |
| ORF1a | P892L, S2193T, L889F, N348S, T224I |  |  |  |  |
| ORF1b | P1763A |  |  |  |  |
| ORF3a | K66*, K67E |  |  |  |  |
| ORF7a | A106S |  |  |  |  |
| ORF14 | V27F |  |  |  |  |
| S | S730C, D1259Y, D1199E, T573I |  |  |  |  |
| N | S180I |  |  |  |  |
|  |  |  |  |  |  |

**Supplemental Table 3**

| **Amino acid mutation** | **Nature of mutation** | **Frequency (%)** |
| --- | --- | --- |
| Q57H | Deleterious | 10 |
| T175I | Neutral | 5 |
| L94F | Neutral | 5 |
| G251V | Deleterious | 5 |
| K16N | Neutral | 5 |
| P25L | Deleterious | 5 |
| W149L | Deleterious | 5 |
| R126T | Deleterious | 5 |
| T176I | Deleterious | 5 |
| L94I | Neutral | 5 |
| A72T | Neutral | 5 |
| T217I | Deleterious | 5 |
| D142N | Deleterious | 5 |
| V90F | Deleterious | 5 |
| Y109C | Deleterious | 5 |
| D155Y | Deleterious | 5 |
| Y156N | Deleterious | 5 |
| K67E | Deleterious | 5 |
| K67* | **_** | 5 |

**Supplemental Table 4**
